# Patients’ Attitudes Toward the Involvement of Medical Students in Their Health Care in the Family Medicine Clinics in the Makkah Region, Saudi Arabia

**DOI:** 10.7759/cureus.42404

**Published:** 2023-07-24

**Authors:** Safa H Alkalash, Ethar H Alhashmi Alamer, Sarah A Munshi, Rawan Aljuwaybiri, Hala T Jawa, Rana K Asiri, Wefag J Sawadi

**Affiliations:** 1 Community Medicine and Health Care, Umm Al-Qura University, Al-Qunfudah, SAU; 2 Family Medicine, Menoufia University, Shebin Alkom, EGY; 3 College of Medicine, Umm Al-Qura University, Makkah, SAU; 4 College of Medicine, Umm Al-Qura University, Al-Qunfudah, SAU

**Keywords:** saudi arabia, medical students, family medicine, communication, attitude

## Abstract

Background

The medical undergraduates in the College of Medicine at Umm Al-Qura University, Saudi Arabia, are dedicated to undergrad training in family health facilities. Throughout this clinical attachment, students receive instructions in family practice, patient management, communication skills, clinical examination, and history-taking.

Objectives

This study was conducted to assess patients' attitudes and key contributing variables toward having medical students participate in their health care consultations in family medicine clinics in the Makkah region of Saudi Arabia.

Methods

A descriptive cross-sectional study was conducted on a sample of 529 patients who attended family medicine clinics in primary health care settings and were living in the Makkah region. The researchers collected the relevant data during a three-month period (from February 1 to the end of April 2023) by administering a validated, well-structured, self-administered online survey of 24 items on several electronic platforms and analyzing it with SPSS Version 23.

Results

The mean of their answers on attitude was found to be 41.6 ± 8.51, suggesting a positive attitude of the patients toward the involvement of medical students in their health care. Around 150 (28.4%) of the participants strongly agreed that medical students obtained their histories, and 119 (22.5%) strongly agreed that medical students performed clinical examinations for them. Around 104 (19.7) of the participants strongly agreed and 140 (26.5) perceived that the involvement of medical students in their health care improves doctors’ competence. Being 56 years old or older, widowed, retired, or having chronic diseases were found to be associated with a more positive attitude of the patients toward the involvement of medical students in their health care, with p-values of 0.024, 0.034, 0.009, and 0.005, respectively.

Conclusions

Patients showed favorable attitudes toward the existence and participation of medical students in their health care in family medicine clinics. The provision of health-related information and the taking of a detailed history were the most notable benefits of medical students’ involvement in patient care, according to most of the patients. Older, widowed, retired, and those with chronic diseases seemed to possess a positive attitude toward the engagement of medical students in their health care in the family medicine clinics.

## Introduction

Family medicine (FM) ranks as one of the most significant medical disciplines in the world since it offers a wide range of continuous, comprehensive, and coordinated health care services to everybody, irrespective of age, gender, or organ or system [1]. A family physician offers primary care to complete families in their communities, deals with physical, mental, and social issues, and organizes comprehensive medical treatments with other health care professionals [2]. In medical education, direct patient discussions have a critical role in the development of clinical reasoning, communication skills, and professional attitudes among medical students, and thus they should use their theoretical knowledge in a practical capacity [3-5]. However, these skills are only effective when both patients and students communicate in constructive, professional ways and have good attitudes about student participation [3]. But not all patients are keen to assist in the education of the medical students. Some patients may view the presence of medical students during a consultation as an invasion of their privacy, in which case they may refuse to have them there [6,7].

In order to meet patients' expectations and improve the standard of the medical treatment they receive, it is important to investigate their perceptions and attitudes concerning their interactions with medical students [8]. Numerous factors, such as the sociocultural and educational backgrounds of the patients, the nature of the examination or procedure, and the characters of the students involved, as regards their gender, seniority level, and behavior, can all affect how comfortable and accepting patients are of medical students helping them with their health care [9,10]. A previous study conducted at two university hospitals in Jeddah, Western Saudi Arabia, found that young and female patients were more likely to have negative attitudes toward medical students' involvement in their care [11]. No previous studies were conducted in Saudi Arabia to evaluate the patients' attitudes toward medical students' involvement in their health care in family health facilities, which represent the front line and health care gate for all populations in disease and health. Therefore, this study aimed to evaluate patients’ attitudes and contributing variables toward medical students' participation in their care in FM clinics in the Makkah region of Saudi Arabia.

## Materials and methods

Study design

The study was designed as a descriptive cross-sectional study to evaluate the attitudes of patients toward having medical students participate in their health care consultations in the FM clinics in the Makkah region of Saudi Arabia, as well as its contributing variables, in the period from February to April 2023. Data were collected based on a well-structured, predesigned questionnaire.

Sample size

The sample size required for this study was calculated using Open Epi Info™ (Centers for Disease Control and Prevention, Atlanta, GA) [12]. Based on a frequency of 51% of Saudi patients having a positive attitude toward involving medical students in their clinical examination and care [11], the total population size in the Makkah region is around 9,000,000, keeping the confidence interval (CI) level at 95% and a 5% margin of error. The minimum number of participants in the sample was determined to be 385.

Study setting

The study was conducted among patients over the age of 15 who attended FM clinics in primary health care settings and were living in the Makkah region, mainly Makkah, Jeddah, Taif, and Al-Qunfudah. Makkah is one of Saudi Arabia's 13 provinces. It is one of the largest provinces in terms of geographical area, with an estimated population of around 9,000,000 people. It is in the historic Hejaz area and boasts a long Red Sea shoreline. Its capital is Makkah, Islam's holiest city, and its major city is Jeddah, Saudi Arabia's primary port city. The province is named after the holy city of Makkah and accounts for 26.29% of Saudi Arabia's population. Makkah region involves 11 governorates, including Jeddah, Rabigh, Ta'if, Qunfudah, Laith, and others.

Data collection tool and procedure

Data were collected from a convenient sample of 529 participants from the Makkah region by using an online questionnaire that was administered to the visitors of patients in primary health care (PHC). The questionnaire was designed by the study researchers based on a review of the literature [3,4,13]. The relevant information was abstracted and organized within the questionnaire that was relevant to the research objective and drafted in Arabic as a 24-item questionnaire with the help of a three-member expert panel, and then it was pre-tested using a pilot study. For the purpose of evaluating language clarity and understandability, 40 replies were tested that constituted around 10% of the calculated sample size. The results of the main study did not contain any of the data from the pilot study, which was simply used to inform the researchers of the survey's validity. Finally, the intended survey's reliability was ensured with Cronbach's alpha coefficient of 0.79.

The questionnaire was categorized into three sections. The first section included questions to evaluate the respondents' socio-demographic data, such as age, gender, marital status, level of education, occupation, monthly income, chronic diseases, and types of chronic illnesses. The second section contained questions to assess whether the medical students have participated in the patient's health care, interviewed them, examined them, and provided them with health-related information. The third section was to evaluate their attitude toward medical students’ participation in their health care in the FM clinics.

Data were collected from all the participants during PHC working hours in the waiting room by disseminating the survey link, which was designed as a Google Form, on different electronic platforms such as WhatsApp, Telegram, and Snap Chat. Also, it was sent to other participants to receive more responses, especially from those who were not attending clinic during the data collection time, to make sure the participants included in our study were asked whether they attended and followed up in PHC; when they answered yes, they proceeded with the questionnaire.

Data analysis

The collected data were entered into Microsoft Excel (Microsoft Corp., Redmond, WA) and analyzed statistically using SPSS Version 23 (IBM Corp., Armonk, NY). Numerical data were presented as mean ± SD, and categorical variables were presented as percentages and frequencies. Attitude statements were expressed on a 5-point Likert scale: 5 = strongly agree, 4 = agree, 3 = neutral, 2 = disagree, and 1 = strongly disagree. A positive attitude of the patient toward the involvement of medical students in their health care in FM clinics was considered when the mean of their answers on attitude statements was 41.6 ± 8.51. Comparisons between groups were made using Student’s t-test and a one-way ANOVA test. The p-value had been deemed significant whenever it was below 0.05.

Ethical considerations and confidentiality

This study was approved by the Umm Al-Qura University Institutional Research Board (IRB) in Makkah under reference number HAPO-02-K-012-2023-02-1461. The confidentiality of the anonymously acquired data was perpetually preserved over time.

## Results

Around 529 participants were included in the current study. Most of them (60.7%) were within the age range of 16 to 25 years. Around 419 (79.2%) were females and 110 (20.8%) were males. Regarding marital status, 347 (65.6%) were single, 156 (29.5%) were married, 20 (3.8%) were divorced, and 6 (1.1%) were widowed. Concerning educational level, 398 (75.2%) were found to be bachelor's degree holders, 101 had a fundamental educational level, and 22 (4.2%) had a higher educational level. Most of the participants (52.4%) were found to be students, 132 (25%) were employees, 102 (19.3%) were unemployed, and 18 (3.4%) were retired. Monthly income was found to be 5,000 SAR or less for around 151 (28.5%) of the participants. In terms of chronic illnesses, only 98 people (18.5%) had at least one. The most frequently reported chronic diseases were found to be diabetes mellitus (9.5%), followed by hypertension (6.2%), asthma (2.1%), cardiovascular diseases (1.5%), and other diseases (4.3%) (Table 1).

**Table 1:** Socio-demographic characteristics of patients and their history of chronic diseases (n=529) SAR, Saudi Arabian Riyal

Variable	Categories	Frequency	Percent
Age (in years)	16–25	321	60.7
	26–35	88	16.6
	36–45	64	12.1
	46–55	35	6.6
	56 and above	21	4
Gender	Male	110	20.8
	Female	419	79.2
Marital status	Single	347	65.6
	Married	156	29.5
	Divorced	20	3.8
	Widowed	6	1.1
Level of education	Illiterate	8	1.5
	Fundamental education	101	19.1
	Bachelor’s	398	75.2
	Higher education	22	4.2
Occupation	Student	277	52.4
	Employee	132	25
	Unemployed	102	19.3
	Retired	18	3.4
Monthly income (SAR)	5,000 or less	151	28.5
	5,000–10,000	115	21.7
	10,000–15,000	143	27
	15,000–20,000	52	9.8
	More than 20,000	68	12.9
Having any chronic illnesses	Yes	98	18.5
	No	431	81.5
Type of chronic illnesses	None	431	81.5
	Hypertension	33	6.2
	Diabetes mellitus	50	9.5
	Cardiovascular diseases	8	1.5
	Hematological diseases	6	1.1
	Asthma	11	2.1
	Other	23	4.3

**Figure 1:**
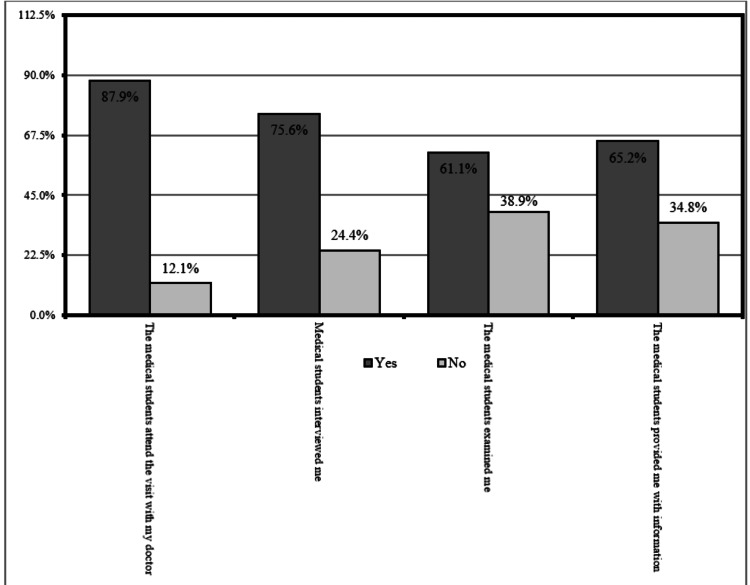
Perceptions of the patients about medical students in the family medicine clinics

Around 465 (87.9%) of patients mentioned that medical students attended the visit with their doctors, 400 (75.6%) of patients reported that medical students interviewed them, 323 (61.1%) patients mentioned that medical students examined them, and 345 (65.2%) patients stated that medical students provided them with health-related information (Figure 1).

The mean of patients' attitudes was 41.6 with a standard deviation of 8.51 (range: 12-60), which was considered a positive attitude. Around 150 (28.4%) of patients strongly agreed that medical students have obtained their histories, and 170 (32.1%) concurred with the same statement. Also, 119 (22.5%) patients strongly agreed that medical students performed a clinical assessment, and 144 (27.2%) agreed with the same statement. Around 145 (27.4%) of patients strongly acknowledged that medical students provided useful information, and 186 (35.7%) agreed with the same item. The medical students behaved in a professional manner, as strongly agreed by 187 (35.3%) of patients; 189 (35.7%) agreed with this statement. Around 231 (43.7%) strongly agreed, and 139 (26.2%) mentioned that seeing medical students is enjoyable. Having medical students participate takes too much time, as strongly accepted by 78 (14.7%) of participants, and 142 (26.8%) agreed with the same statement. Also, 59 (11.2%) strongly agreed and 98 (18.5%) agreed that having medical students involved interferes with their relationship with their doctors. Having medical students in the clinic makes patients feel good, and this was strongly agreed on by 107 (20.2%) of patients; 130 (24.6%) agreed on the same statement. Around 136 (25.7%) strongly agreed that they shared their disease information with the doctor in the presence of medical students, and 151 (28.5%) agreed with the same statement (Table 2).

**Table 2:** Attitudes of patients toward medical students in family medicine clinics

Statement	Strongly agree	Agree	Neutral	Disagree	Strongly disagree
	n (%)				
The medical students obtained history	150 (28.4)	170 (32.1)	127 (24)	33 (6.2)	49 (9.3)
The medical students performed a clinical examination	119 (22.5)	144 (27.2)	149 (28.2)	56 (10.6)	61 (11.5)
The medical students provided useful information	145 (27.4)	186 (35.2)	112 (21.2)	37 (7)	49 (9.3)
The medical students behaved in a professional manner	187 (35.3)	189 (35.7)	112 (21.2)	18 (3.4)	23 (4.3)
Seeing the medical students in the clinic is enjoyable	231 (43.7)	139 (26.3)	91 (17.2)	29 (5.5)	39 (7.4)
Involvement of medical students takes too much time	78 (14.7)	142 (26.8)	181 (34.2)	67 (12.7)	61 (11.5)
Involvement of medical students interferes with the doctor-patient relationship	59 (11.2)	98 (18.5)	137 (25.9)	127 (24)	108 (20.4)
Medical students’ participation decreases time with the doctor	68 (12.9)	88 (16.6)	151 (28.5)	121 (22.9)	101 (19.1)
Involvement of medical students improves doctor’s competence	104 (19.7)	140 (26.5)	183 (34.6)	59 (11.2)	43 (8.1)
Involvement of medical students improves the quality of health care	135 (25.5)	161 (30.4)	165 (31.2)	40 (7.6)	28 (5.3)
Presence of medical students in the clinic makes a patient feels good	107 (20.2)	130 (24.6)	177 (33.5)	68 (12.9)	47 (8.9)
Patients share their disease information with the doctor in the presence of medical students	136 (25.7)	151 (28.5)	115 (21.7)	65 (12.3)	62 (11.7)

Age, marital status, occupation, and having chronic diseases were variables that significantly affected the patients' attitudes, as being 56 years old or older, widowed, retired, and having chronic illnesses all tended to have a favorable attitude compared to others, with p-values of 0.024, 0.034, 0.009, and 0.005, respectively. Gender, educational level, and monthly income were not found to significantly affect the patient's attitude (p-values = 0.920, 0.115, and 0.121, respectively) (Table 3).

**Table 3:** Association between socio-demographic data and attitudes of patients toward medical students in family medicine clinics SAR, Saudi Arabian Riyal; SD, standard deviation A p-value of <0.05 is statistically significant

Variable	Categories	Attitude		P-value
		Mean	SD	
Age (in years)	16–25	41.1	8.47	0.024
	26–35	40.7	9.04	
	36–45	42.7	8.24	
	46–55	44.0	6.40	
	56 and above	45.7	9.14	
Gender	Male	41.7	8.78	0.920
	Female	41.6	8.45	
Marital status	Single	40.8	8.76	0.034
	Married	43.1	7.74	
	Divorced	42.2	9.47	
	Widow	45.2	3.37	
Level of education	Illiterate	45.3	7.50	0.115
	Fundamental education	42.9	7.71	
	Bachelor’s	41.3	8.56	
	Higher education	39.5	10.65	
Occupation	Student	40.9	8.54	0.009
	Employee	42.1	8.23	
	Unemployed	41.5	8.51	
	Retired	47.7	7.93	
Monthly income (SAR)	5,000 or less	41.0	8.35	0.121
	5,000–10,000	42.4	8.59	
	10,000–15,000	42.5	8.47	
	15,000–20,000	39.2	8.25	
	More than 20,000	41.4	8.78	
Having any chronic illnesses	Yes	43.7	8.28	0.005
	No	41.1	8.49	

## Discussion

The determination of the exact patient's perceptions of medical students in clinics is of considerable significance as it demonstrates the comprehension of patients regarding the role of medical students in clinics and the importance of taking a proper history, performing an examination, and providing information [14]. The aim of the current study was to assess patients’ attitudes and contributing variables toward the involvement of medical students in their health care in FM clinics. Less than two-thirds (60.7%) of the respondents were within the age group of 16-25 years, and more than two-thirds (79.2%) of the participants were female. Concerning educational level, the majority (75.2%) of participants were found to be bachelor's degree holders. Half of the participants (52.4%) were students. Regarding chronic illnesses, only 18.5% had such diseases. The most frequently reported chronic diseases were diabetes mellitus, as reported by 9.5% of the participants, followed by hypertension, which was found in 6.2% of the participants. The vast majority (87.9%) of patients mentioned that medical students attended the visit with their doctors. Less than two-thirds (61.1%) mentioned that medical students examined them, and around two-thirds (65.2%) of them stated that medical students provided them with health-related information. Similar findings were reported in the congruent study conducted by Taha et al., in which around 80% of patients were observed and examined by medical students [8]. This impressive finding refers to the positive role of medical undergraduates in workplace settings as active trainers, which is one of the teaching strategies that improve medical students’ competencies, as any patient prefers to have doctors who can conduct effective communication, diagnose the disease correctly, and treat it successfully [13].

The mean attitude of patients toward the involvement of medical students in their health care was 41.6, which is considered a positive attitude, and this was consistent with the findings of the parallel studies conducted by Mwaka et al. and by Malhotra and Hosdurga, in which the vast majority had a positive perception about medical students’ involvement in their treatment [3,15]. Less than one-third (28.4%) of patients strongly agreed with the medical student taking their medical history, and 144 (27.2%) agreed on the same statement. Analogous findings were reported in the studies, in which more than two-thirds of the participants accepted and mentioned the importance of history-taking by medical students [3,16]. Indeed, this research has indicated that medical students behaved in a professional manner; this was strongly agreed upon by more than one-third (35.3%) of patients, and another third (35.7%) just agreed; this was found to be consistent with the results obtained in the congruent study conducted by Ghobain et al., in which 87% of the participants agreed that medical students had dealt with them with professionalism [17]. Having medical students participate takes too much time, as strongly agreed on by 14.7% of patients. Around 11.2% of the participants strongly agreed and 18.5% of the participants agreed that having medical students involved interferes with the doctor-patient relationship. Around 14.7% of the participants strongly agreed and 26.8% agreed that having medical students participate prolongs their consultation time; similar results were noted in the study conducted by Laiq-Uz-Zaman Khan et al., in which 43% felt it resulted in prolonging waiting time due to teaching [18]. Around one-quarter (25.5%) of patients strongly agreed and less than one-third (30.4%) agreed that having medical students involved improved the quality of care they received; this was also proportionate to what was reported in Onotai et al.’s study [19]. Having medical students in the clinic makes patients feel good, and this was strongly agreed on by 20.2% of the participants, and 24.6% just agreed on the same statement. Consistent results were obtained in the corresponding study conducted by Hartz and Beal and by Devera-Sales et al., in which most of the participants felt good about the presence of medical students during their health care consultation [20,21].

Regarding the association between socio-demographic characteristics and the patients' attitudes, age was found to be significantly associated with attitude, as being 56 years old or older was linked with a more positive attitude, which is supported by the finding extracted by Aljoudi et al. that young patients did not accept the presence of medical students during their clinical consultation [11]. Gender, educational level, and monthly income were not found to significantly affect the patients' attitudes, and this was contradictory to the findings of the study conducted by Menezes et al., in which educational level significantly influenced the patient's attitude [22]. Additionally, a previous Saudi study in the western region found that females and young patients had negative attitudes regarding the presence of medical students during their clinical consultation [11]. The difference between these studies’ findings may be related to the discrepancy in the studies’ settings and samples, where the old ones were conducted in teaching hospitals and not in FM clinics.

Strengths and limitations of the study

The strengths of this study include the large sample size of 529 patients, which reflects the patients’ attitudes toward the involvement of medical students in their care in FM clinics and the fact that previous studies on such an important issue are absent in FM clinics in Saudi Arabia. The current study's findings might help medical schools evaluate their own patient-student interactions. The study's unique points of investigation included forms of student-patient interaction not previously explored in local studies, such as medical students' professionalism, respect for patient privacy, and confidence. This study, however, has numerous drawbacks. Because it was cross-sectional research, there was a possibility of bias in reporting results, and cause and effect had not been considered. Furthermore, the online nature of data collection may limit the collection to educated participants only; therefore, we recommend further research on this issue using directed interviews.

## Conclusions

The patient showed a more positive attitude toward the involvement of medical students in their health care in family health clinics. The provision of detailed health-related information and the taking of detailed histories were the most notable benefits believed by patients. Few patients who were concerned about time spent and their relationship with the doctor expressed a negative attitude towards medical students in FM clinics. Older, widowed, retired, and chronic disease patients had a more positive attitude than others. More description of the medical student's role and position in the FM clinic is needed to demonstrate the exact role and importance of the medical student's work to patients attending FM clinics. This will result in a better understanding and more cooperative environment and facilities, as well as positive relationships between patients and medical students, and hence more accurate information that will aid in reaching a diagnosis and treatment.
